# The Loss of Children

**Published:** 1863-11

**Authors:** 


**The Loss of Children.**—Those who have never passed through this fiery furnace, which tries the inmost heart, can not sympathize with bereaved parents whose hearts bleed over their children dead. To describe the anguish which rends their hearts, as they gaze upon the loved forms on whom their fondest hopes and highest aspirations had rested so firmly, now cold and lifeless in their coffin-home, would require a pen dipped in the very essence of the sublimest sorrow itself. None but the parents can feel it, and none but those who have mourned like them, can sympathize with those who mourn the death of their children. The loss no power on earth can make good or even alleviate. No power on earth can bring them back, and place them again beneath their parents' loving gaze and fond care. From earth they have taken their final departure, never, never to return. The little chair they occupied, the little plate and the knife and fork they used, will be to them of service no more—but merely lonely mementoes of their existence. The patter of their little feet upon the floor, and the music of their sweet, sweet voices, will greet the parents' ear never again on earth. All will be a recurrence of all that is dreary and dismal. But hope, plumed by religion, points to a happy meeting in another and better world.

